# Yang-warming method in the treatment of diabetic peripheral neuropathy: an updated systematic review and meta-analysis

**DOI:** 10.1186/s12906-017-1927-5

**Published:** 2017-08-25

**Authors:** Sharad Panthi, Xirun Jing, Chenghan Gao, Tianshu Gao

**Affiliations:** Department of Chronic Disease, Liaoning University of Traditional Chinese Medicine, Chongshan East Road No.72, Shenyang, Liaoning 110032 China

**Keywords:** Yang-warming method, Yang-warming Chinese medicine, Diabetic peripheral neuropathy, Meta-analysis

## Abstract

**Background:**

Various studies have suggested the effectiveness of Chinese medicine in the treatment of diabetic peripheral neuropathy (DPN). There are several principles and methods in Chinese medicine for the treatment of DPN and yang-warming method is one of them. The purpose of this meta-analysis was to review the effectiveness and safety of yang-warming method using yang-warming Chinese medicine (YCM) in the treatment of DPN.

**Methods:**

A computer-based search of the articles from January 2001 to April 2016 with Chinese and English databases such as CNKI, CBM, Wanfang, VIP, Medline, Embase and Cochrane central register of controlled trials as well as manual search of the related articles was conducted. Randomized Controlled Trials (RCTs) comparing yang-warming Chinese medicines with western medicines in the treatment of DPN were considered for the study. The outcome measures were change in the sensory or motor nerve conduction velocity, total efficacy rate evaluated by clinical symptoms improvement, and adverse events. Two authors independently assessed the methodological quality of the included articles using Jadad scale and the twelve criteria recommended by Cochrane Back Review Group. Data were analyzed using RevMan 5.3 software provided by Cochrane collaboration.

**Results:**

A total of 25 articles were taken for the study. Meta-analysis results showed that yang-warming Chinese medicines used in the formula alone or in combination with western medicines improved the nerve conduction velocity (NCV) in comparison to western medicines alone (*p* < 0.001). There was also a significant difference in the total efficacy rate between the two groups (*p* < 0.001). Most of the included studies did not clearly report the adverse events.

**Conclusions:**

Yang-warming Chinese medicines alone or in combination with western medicines were apparently better than conventional western medicines in the treatment of DPN. Because of the poor quality of the reported works that were available for the present meta-analysis, it is earlier to claim the superiority of yang-warming method using YCM to western medicines for the treatment of DPN. To support these early findings, further standardized and rigorous RCTs are required.

## Background

In recent years, the global incidence of diabetes has a rising trend. Diabetes is an important risk factor for cardiovascular and cerebrovascular diseases. Diabetes in course of time results in various macrovascular and microvascular complications. Diabetic peripheral neuropathy (DPN) is the most common symptomatic complication affecting up to 50% of patients with diabetes [1]. According to the EURODIAB IDDM Complications Study, the prevalence rate of DPN across Europe was 28% [2]. According to a study by Gordois et al. [3], the annual cost of DPN and its complications was 4.6–13.7 billion dollar in the US in 2001. Up to 50% of DPN may be asymptomatic and patients are at greater risk of insensate injury, foot ulcer, and ultimate amputation [4]. DPN causes great physical and mental sufferings to the patients and their families, which in turn seriously affects the quality of life and life span of the patients.

The pathophysiology of DPN is considered to be complex and multifactorial. A number of risk factors are associated with DPN, poor glycemic control and duration of diabetes mellitus (DM) being the prime factors. Other factors associated are genetic susceptibility, low HDL, cardiovascular diseases, and life style factors such as alcohol and smoking. Both metabolic and ischemic factors play an important role in DPN. Alteration of the polyol pathway, increased advanced glycosylated end products (AGEs) formation and increased oxidative stress may lead to nerve dysfunction [5, 6]. AGEs might damage nerve fibers due to an effect on matrix metalloproteinases. [7]. The overall management of DPN patients is now mainly focused on glycemic control and addressing cardiovascular risks in addition to managing painful symptoms [8]. Glycemic control is the main stay of treatment and management is directed to achieve near- normal glycemia [9, 10]. So far, there is no definitive treatment for painful DPN. Some of the useful drugs in the treatment of DPN are antidepressants, anticonvulsants, phenothiazines, calcitonin, local anesthetics, NSAIDS, and steroids, which have their own limitations. The use of vitamin B12 and alpha-lipoic acid has also been found to be effective in the management of DPN [11, 12]. Additionally, the DPN management plans have also focused on blood pressure control, dyslipidemia, and lifestyle modifications.

Chinese herbal medicine has been widely used in China for thousands of years for the management of DM and DPN-like conditions. Traditional Chinese medicine (TCM), which includes Chinese herbs, acupuncture, massage and other classical methods of treatments, is proven to be effective in the treatment of DPN [13]. Various studies including both animal and bed side experiments have verified the efficacy of TCM regarding the management of DPN. The evidences from the clinical research have suggested that Chinese herbs possibly reduce oxidative stress through Nrf2 and Bcl2 [14], alternate autonomic nerve damage [15], and decrease neuropathic pain [16]. Chinese herbs are likely to have certain effects on the promotion of NGF- expression and insulin-like growth factor (IGF) [17, 18]. Some studies have also reported that Chinese herbs may help in nerve regeneration [19], and promote blood microcirculation and nourish peripheral nerves [20]. The other possible pathways for the action of Chinese herbs in preventing DPN could be alteration of polyol pathway [21], reduction in the formation of AGEs [22], and activation of protein kinase C [23, 24]. The main basis of treatment in TCM is syndrome differentiation. According to syndrome differentiation, TCM has different treatment principles for DPN such as nourishing yin and blood, regulating the qi-movement, activating blood and thus removing blood stasis, clearing channels, warming yang and thus dispelling cold, and invigorating the kidney and spleen. The use of herbs also differs according to these principles. In recent years, there have been many studies using yang-warming Chinese medicine (YCM) for the treatment of DPN. According to the TCM theory, yang deficiency is one of the most important factors in the pathogenesis of DPN and thus yang-warming principle is considered to be the key treatment principle [25]. However, due to variation in the sample size and methodological quality of the studies, the efficacy and safety of this method is still not fully understood. Although there have been a few systematic reviews and meta-analyses in recent years to evaluate the efficacy and safety of Chinese herbs for DPN, these studies didn’t differentiate the categories of the herbs used [26–29]. Understanding the treatment principles of TCM, however, is not conclusive without appropriate categorization of the herbs. Moreover, non-TCM experts may face problems to perform further research in the related field. Therefore, we conducted the present meta-analysis to review the efficacy and safety of the yang-warming method for the treatment of DPN taking into account the herb categories and thus the treatment principles.

## Methods

For this review, we followed the current practices for conducting systematic reviews and was completed according to the PRISMA guidelines for the reporting of systematic reviews and meta-analyses [30, 31].

### Data sources and information retrieval

The clinical randomized controlled trials (RCTs) from January 2001 to April 2016 which used yang warming Chinese medicines were selected. The 7 Chinese and English databases named CNKI, CBM, Wanfang, VIP, Medline, Embase and Cochrane central register of controlled trials were searched using keywords diabetic peripheral neuropathy or diabetic neuropathy or DPN and Chinese medicine or herbs or yang warming Chinese herbs or TCM. The similar search strategy with Chinese terms was applied while searching Chinese databases. At the same time, we manually searched related articles and references.

### Inclusion criteria

#### Type of study

Randomized controlled trials published in both English and Chinese languages regardless of whether there is single blind, double blind, triple blind or non-blind.

#### Study objects

Clearly diagnosed patients of diabetic peripheral neuropathy.

A) Diagnostic criteria for diabetes: According to the 1999 World Health Organization (WHO) diagnostic criteria for diabetes or the 1997 American Diabetes Association (ADA) diagnostic criteria for diabetes or with reference to the Guidelines for Clinical Research of Traditional Chinese Medicine (2002 edition).
B) Criteria for the diagnosis of diabetic peripheral neuropathy: A reference to the WHO international collaborative research of the diabetic peripheral neuropathy (WHOPNTF).

#### Interventions

Usually conventional treatment, combined with the following treatments.

A) The treatment group was treated with yang-warming Chinese medicine, and the control group was treated with western medicine alone. At the same time both groups were given conventional treatment to control blood glucose.
B) The treatment group was treated with yang-warming Chinese medicine combined with Western medicine, and the control group was treated with western medicine alone. While two groups were given conventional treatment to control blood glucose.
C) Yang-warming Chinese medicines were self-made decoction and proprietary Chinese medicines.
D) The types of western medicine were western conventional medicines used in the treatment of DPN.

### Exclusion criteria

A) Clinical trials with other methods of treatment as intervention measures. B) Non-random or uncontrolled trials; C) Neurological symptoms and signs associated with other neurological diseases; D) Case reports, expert experiences and reviews; E) Non-clinical studies like animal experiments or pharmacokinetics studies etc. E) Trials using acupuncture and external yang-warming Chinese medicines/methods.

### Outcome measures

A) Nerve conduction velocity (NCV): including the peroneal nerve, tibial nerve and median nerve, in units of m/s; B) Total efficacy rate: clinical efficacy was categorized as effective yang- warming Chinese medicines and ineffective yang- warming Chinese medicines. The ability of Chinese medicines to improve clinical symptoms and signs of neuropathy were taken as effective and those unable to reduce the clinical symptoms and signs or got even worsed were taken as ineffective.

### Publication bias analysis

The publication bias was analyzed by Funnel plot. Funnel plot is a common method of measuring a qualitative publication bias. If the funnel plot asymmetrical on both sides of the distribution, suggesting the presence of bias; if symmetrical on both sides of the funnel plot, suggesting no bias.

### Sensitivity analysis

In the process of meta-analysis when reviewer encounters small sample size should perform sensitivity analysis in order to avoid bias. So we also performed sensitivity analysis.

### Selection of studies

A) Reading the title and abstract of each article, articles not meeting the inclusion criteria and duplicate publications were removed; B) Full text search for potential relevant literatures; C) according to the inclusion and exclusion criteria studies were included in the review; D) For information of incomplete reporting, original authors were tried to contact for supplementary relevant information; E) Two reviewers independently did the quality assessment and cross checking, discrepancies between two authors was resolved by discussion or consultation with third reviewer; F) Finally data were extracted from selected articles.

### Quality assessment of included articles

Randomized controlled trials are the “gold standard” in the design of experimental study, currently meta- analysis is the mostly used quality evaluation tool for them. The methodological quality of the included studies was accessed by Jadad scale [32] and by using the twelve criteria recommended by the Cochrane Back Review Group [33]. Studies having the scores of 1 or 2 points were considered low quality and 3–5 points as high quality in Jadad scale. Similarly, in 12- point criteria, studies scoring at least 6 points out of 12 points were considered as having a low risk of bias.

### Statistical analysis

The data were analyzed by using Revman 5.3 software provided by Cochrane Collaboration. At first heterogeneity test was done, significant difference for heterogeneity test was considered when calculated I^2^ > 50% value, if showed no difference, fixed –effect model was used otherwise random-effect model was used. For continuous data, weighted mean difference/mean difference (WMD/MD) or standardized mean difference (SMD) with 95% confidence interval (CI) was calculated. If dichotomous data available, odds ratios with 95% CI were calculated. The Z test was used to compare the overall effects of treatment group and control group, and differences were considered to be statistically significant when *p* < 0.05.

## Results

### Study selection and characteristics of included studies

**Fig. 1:**
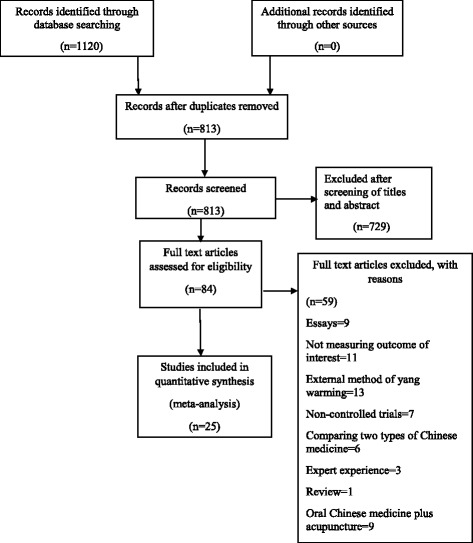
Study selection flow diagram

A total of 1120 articles were retrieved, and 25 articles were selected according to the inclusion criteria and exclusion criteria [34–58]. Full text of finally screened articles were carefully read. The study selection process is summarized in a flow diagram (Fig. 1). All studies included were conducted in China and were published in Chinese language. A total of 1203 patients were included in the group comparing yang-warming medicines with pure western medicines (Vitamin B12/ Vitamin B12 + B6/VitaminB12 + B6 + NGF); treatment group (yang-warming medicine) 632 cases, and control group (western medicines) 571 cases with treatment course of 20 days to 3 months. A total of 1068 patients were included in the group comparing yang-warming Chinese medicine plus western medicine (Vitamin B12/Vitamin B12 + B1/Vitamin B12 and/or α-Lipoic acid) with pure western medicine (Vitamin B12/Vitamin B12 + B1/Vitamin B12 and/or α-Lipoic acid); treatment group (yang-warming Chinese medicine combined with western medicine) 540 cases, and control group (Western medicine) 528 cases with treatment course ranging from 30 days to 4 months. In the course of the study, patients had good compliance. The general information about all studies is shown in Table 1. A total of 2271 patients were included in the study. All the studies were referred to use randomization but only 5 studies, Chen 2014 [34]; Cao 2012 [45]; Zhang 2007 [43]; Liu 2015 [52], and Ma 2015 [55] explicitly used random number table method for randomization, and rest of the studies, although used randomization for grouping, but were not referred to use of any specific methods. None of the included studies mentioned allocation concealment, blinding and withdrawal or dropouts. There was no statistical difference in age, sex and disease duration in all the studies and were comparable. Among the studies comparing yang-warming Chinese medicine with western medicine alone (Vitamin B12/Vitamin B12 + B6/Vitamin B12 + B6 + NGF), 10 articles described peroneal nerve conduction velocity [39, 40, 44–47, 50–53]; 5 articles described tibial nerve conduction velocity [39, 42, 45, 48, 53]; 6 articles described median nerve conduction velocity [39, 45, 46, 48, 51, 52] and 15 articles described clinical effectiveness of the chinese medicine [39–53]. Among the studies comparing yang warming Chinese medicine plus western medicine (Vitamin B12/Vitamin B12 + B1/Vitamin B12 and/or α-Lipoic acid) with western medicine (Vitamin B12/Vitamin B12 + B1/Vitamin B12 and/or α-Lipoic acid) alone, 8 articles compared peroneal nerve conduction velocity [34–36, 38, 54, 55, 57, 58]; 3 articles compared tibial nerve conduction velocity [34, 37, 56]; 5 articles compared median nerve conduction velocity [35, 36, 54, 57, 58], and 10 articles described clinical effectiveness[34–38, 54–58].

**Table 1:** General characteristics of the included studies

First author	DM/DPN duration (years)		Age (years)		Sex (M/F)		Sample size		Intervention		Course of treatment	Adverse events	Main outcome measures
	T	C	T	C	T	C	T	C	T	C			
Chen 2014 [34]	1/12–10	1/6–8	53.4 ± 14.8	52.9 ± 14.5	21/21	20/18	42	38	J + H + Vitamin B12	J+ Vitamin B12	4 weeks	n.r.	NCV, TER
Qi 2010 [35]	(103.24 ± 17.62)/12	(98 ± 20.9)/12	56.31 ± 7.4	59.7 ± 8.1	27/15	25/15	42	40	J + H + Vitamin B12	J+ Vitamin B12	4 weeks	n.r.	NCV, TER
Li 2001 [36]	(37 ± 6)/12	(36 ± 6)/12	60 ± 12	62 ± 8	17/14	12/19	31	31	J + H + Vitamin B12	J+ Vitamin B12	4 weeks	No	NCV, TER
Chen 2007 [37]	10.8	10.2	55.2 ± 5.4	56.3 ± 6.1	20/21	19/22	41	41	J + H + Vitamin B12 & B1	J+ Vitamin B12 & B1	30 days	0/1	NCV, TER
Lin 2010 [38]	7.0 ± 2.3	7.2 ± 2.2	56.7 ± 1.8	54.9 ± 6.5	52/52	58/41	104	99	J + H + Vitamin B12	J+ Vitamin B12	80 days	n.r.	NCV, TER
Qiao 2006 [39]	n.r.	n.r.	n.r.	n.r.	n.r.	n.r.	48	42	J + H	J+ Vitamin B12	20 days	No	NCV, TER
Liu 2006 [40]	7.3	8.31	58.2	58.4	17/30	16/30	47	46	J + H	J+ Vitamin B12	8 weeks	n.r.	NCV, TER
Hua 2009 [41]	8.2	7.5	58.45	60.65	12/20	18/13	32	31	J + H	J+ Vitamin B12	30 days	n.r.	TER
Cao 2012 [42]	n.r.	n.r.	55.8 ± 4.2	54.7 ± 3.3	35/31	28/26	66	54	J + H	J+ Vitamin B12	8 weeks	5/2	NCV, TER
Zhang 2007 [43]	7.93 ± 2.36	6.88 ± 2.40	56.81 ± 9.36	55.64 ± 9.73	13/17	16/14	30	30	J + H	J+ Vitamin B12	8 Weeks	n.r.	TER
Hong 2006 [44]	7.72 ± 4.01	7.62 ± 3.80	58.22 ± 6.31	56.83 ± 7.02	20/10	18/12	30	30	J + H	J+ Vitamin B12, B1 and NGF	80 days	No	TER
Chen 2005 [45]	2.32 ± 1.50	2.34 ± 1.21	51.20 ± 7.81	52.3 ± 8.52	28/17	18/12	45	30	J + H	J+ Vitamin B12 and B6	4 weeks	n.r.	NCV, TER
Lin 2011 [46]	11.63 ± 5.09	11.5 ± 5.98	61.85 ± 8.09	62.7 ± 8.11	35/25	18/12	60	30	J + H	J+ Vitamin B12	3 months	n.r.	NCV, TER
Yi 2008 [47]	11.06 ± 3.67	11.13 ± 3.59	59.87 ± 6.37	60.03 ± 6.46	16/14	15/15	30	30	J + H	J+ Vitamin B12	4 weeks	n.r.	NCV, TER
Li 2012 [48]	8.4	7.6	56.5	54.8	35/25	38/22	60	60	J + H	J+ Vitamin B12	4 weeks	No	NCV, TER
Chen 2009 [49]	11.16 ± 3.57	10.93 ± 4.01	59.67 ± 6.27	60.03 ± 6.42	16/14	15/15	30	30	J + H	J+ Vitamin B12	12 weeks	n.r.	TER
Huang 2015 [50]	6–20	5–19	41–79	40–70	30/30	40/20	60	60	J + H	J+ Vitamin B12	4 weeks	n.r.	NCV, TER
Song 2015 [51]	(52.3 ± 41.3)/12	(54.3 ± 36.7)/12	59.8 ± 6.3	59.2 ± 9.4	14/16	13/17	30	30	J + H	J+ Vitamin B12	12 weeks	n.r.	NCV, TER
Chen 2015 [52]	6.91 ± 5.57	6.30 ± 4.97	54.32,61.07	54.38,62.27	14/16	17/13	30	30	J + H	J+ Vitamin B12	60 days	n.r.	NCV, TER
Zhao 2015 [53]	7.60 ± 5.8	7.11 ± 4.90	57.41 ± 8.98	55.83 ± 9.64	22/18	21/19	40	40	J + H	J+ Vitamin B12	8 weeks	3/4	NCV, TER
Liu 2015 [54]	6.44 ± 2.87	6.34 ± 2.78	53.15 ± 10.23	52.25 ± 10.18	34/31	35/29	65	64	J + H + α-lipoic acid	J+ a-lipoic acid	4 weeks	1/3	NCV, TER
Ma2015 [55]	8.5 ± 2.9	8.8 ± 2.7	62.1 ± 6.7	61.8 ± 6.9	26/17	28/15	43	43	J + H + α-lipoic acid	J+ a-lipoic acid	4 weeks	n.r.	NCV, TER
Chen 2015 [56]	12.62 ± 3.17	11.26 ± 3.51	60.16 ± 5.71	61.4 ± 6.15	17/13	15/15	30	30	J + H + Vitamin B12	J+ Vitamin B12	4 months	No	NCV, TER
Zhang 2015 [57]	8.9 ± 2.1	9.1 ± 2.1	53.7 ± 2.3	54.2 ± 2.5	41/19	39/21	60	60	J + H + Vitamin B12	J+ Vitamin B12	4 weeks	1/0	NCV, TER
Lang 2015 [58]	10.72 ± 7.96	11.34 ± 8.25	58.75 ± 19.01	57.02 ± 18.53	46/36	52/30	82	82	J + H + Vitamin B12 + α-lipoic acid	Vitamin B12 + a-lipoic acid	2 weeks	n.r.	NCV, TER

*T* Treatment group, *C* control group, *M* Male, *F* Female, *J* Basic treatment given in each group, *H* Yang warming Chinese medicine, *NCV* nerve conduction velocity, *TER* Total efficacy rate, *No* no adverse events, *n.r.* not reported

### Quality of the included studies

All the included studies were of low methodological quality scoring 1 to 2 in Jadad scale and 3–5 in 12- point criteria. The details of the methodological quality of the studies is shown in Table 2.

**Table 2:** Quality of the included trials

Study ID	12-item criteria													Jadad scale					
	A	B	C	D	E	F	G	H	I	J	K	L	T	a	b	c	d	e	T
Chen 2014 [34]	+	−	−	−	−	−	−	?	+	+	?	+	4	1	1	0	0	0	2
Qi 2010 [35]	−	−	−	−	−	−	−	?	+	+	?	+	3	1	0	0	0	0	1
Li 2001 [36]	−	−	−	−	−	−	−	?	+	+	+	+	4	1	0	0	0	0	1
Chen 2007 [37]	−	−	−	−	−	−	−	?	+	+	+	+	4	1	0	0	0	0	1
Lin 2010 [38]	−	−	−	−	−	−	−	?	+	+	?	+	3	1	0	0	0	0	1
Qiao 2006 [39]	−	−	−	−	−	−	−	?	+	+	+	+	4	1	0	0	0	0	1
Liu 2006 [40]	−	−	−	−	−	−	−	?	+	+	?	+	3	1	0	0	0	0	1
Hua 2009 [41]	−	−	−	−	−	−	−	?	+	+	?	+	3	1	0	0	0	0	1
Cao 2012 [42]	+	−	−	−	−	−	−	?	+	+	+	+	5	1	1	0	0	0	2
Zhang 2007 [43]	+	−	−	−	−	−	−	?	+	+	?	+	4	1	1	0	0	0	2
Hong 2006 [44]	−	−	−	−	−	−	−	?	+	+	+	+	4	1	0	0	0	0	1
Chen 2005 [45]	−	−	−	−	−	−	−	?	+	+	?	+	3	1	0	0	0	0	1
Lin 2011 [46]	−	−	−	−	−	−	−	?	+	+	?	+	3	1	0	0	0	0	1
Yi 2008 [47]	−	−	−	−	−	−	−	?	+	+	?	+	3	1	0	0	0	0	1
Li 2012 [48]	−	−	−	−	−	−	−	?	+	+	+	+	4	1	0	0	0	0	1
Chen 2009 [49]	−	−	−	−	−	−	−	?	+	+	?	+	3	1	0	0	0	0	1
Huang 2015 [50]	−	−	−	−	−	−	−	?	+	+	?	+	3	1	0	0	0	0	1
Song 2015 [51]	−	−	−	−	−	−	−	?	+	+	?	+	3	1	0	0	0	0	1
Chen 2015 [52]	−	−	−	−	−	−	−	?	+	+	?	+	3	1	0	0	0	0	1
Zhao 2015 [53]	−	−	−	−	−	−	−	?	+	+	+	+	4	1	0	0	0	0	1
Liu 2015 [54]	+	−	−	−	−	−	−	?	+	+	+	+	5	1	1	0	0	0	2
Ma 2015 [55]	+	−	−	−	−	−	−	?	+	+	?	+	4	1	1	0	0	0	2
Chen 2015 [56]	−	−	−	−	−	−	−	?	+	+	+	+	4	1	0	0	0	0	1
Zhang 2015 [57]	−	−	−	−	−	−	−	?	+	+	+	+	4	1	0	0	0	0	1
Lang 2015 [58]	−	−	+	−	−	−	−	?	+	+	?	+	4	1	0	0	0	0	1

A to L: the 12- item criteria. A: adequate method of randomization, B: allocation concealment, C: patient blinding, D: care provider blinding, E: outcome assessor blinding, F: incomplete outcome data addressed (IIT analysis), G: incomplete outcome data addressed (dropouts), H: free of selective outcome reporting, I: baseline similarity, J: co-interventions constant, K: compliance acceptable, L: timing of the outcome assessment

a to e: the Jadad scale. Points were awarded as follows: a = the study was described as randomized, 1 point; b = randomization method was appropriate, 1 point; c = the study was described as double blind, 1 point; d = the blinding method was appropriate, 1 point; e = description of withdrawals and dropouts, 1 point. The score of Jadad scale ranges from 1 to 5, higher the score, better the quality of the study. *T* total score

### Yang- warming Chinese herbs

Twenty-three yang-warming Chinese medicine formulas were used in 25 included studies. All were internally used medicines in the form of decoction or pills or capsules. The details of the yang-warming Chinese herbs in the formulas are shown in Table 3.

**Table 3:** Yang-warming Chinese herbs included in the studies

Study ID	Name of the formula	Yang-warming herbs used in the formula	Usage
Chen 2014 [34]	Wenyangbushen Tang (Decoction)	Rougui (*Cinnamomum cassia* bark), Fuzi (Aconitum carmichaeli root), Shanzhuyu (Cornusofficinalis)	100 ml BID
Qi 2010 [35]	Yiqiwenyangtongluo Tang (Decoction)	Guizhi (Cinnamomum cassia twig), Xixin (Asarum heterotropoidesrhizhome)	200 ml BID
Li 2001 [36]	YiqiyangyinwenyangHuoxue Tang (Decoction)	Guizhi (Cinnamomum cassia twig), Fuzi (Aconitum carmichaeli root),Ganjiang (*Zingiber officinale*), Shanzhuyu (Cornusofficinalis)	200 ml BID
Chen 2007 [37]	Yiqiwenyanghuoxue Tang (Decoction)	Guizhi (Cinnamomum cassia twig), Fuzi (Aconitum carmichaeli root)	_
Lin 2010 [38]	Yiqiwenyanghuoxuetongluo Tang (Decoction)	Guizhi (Cinnamomum cassia twig), Xixin (Asarum heterotropoidesrhizhome)	_
Qiao 2006 [39]	Wenyanghuoxuetongluo Tang (Decoction)	Guizhi (Cinnamomum cassia twig), Xixin (Asarum heterotropoidesrhizhome), Rougui (Cinnamomum cassia bark), Buguzhi (Psoraleacorylifolia fruit)	_
Liu 2006 [40]	Wenyangqushitongluo Tang (Decoction)	Fuzi (Aconitum carmichaeli root), Rougui (Cinnamomum cassia bark),Paojiang (dried Zingiber officinale), Mahuang (*Ephedra sinica* stem), Lujiaojiao (*Cervus Nippon* horn)	_
Hua 2009 [41]	Wenyangtongluo Tang (Decoction)	Mahuang (Ephedra sinica stem),Guizhi(Cinnamomum cassia twig)	100 ml BID
Cao 2012 [42]	TongluoJiaonang (Capsule)	Guizhi (Cinnamomum cassia twig), Xixin (Asarum heterotropoidesrhizhome)	6 capsules TID
Zhang 2007 [43]	Yiqihuoxuewenyang Tang (Decoction)	Fuzi (Aconitum carmichaeli root), Mahuang (Ephedra sinica stem), Xixin (Asarum heterotropoidesrhizhome), Guizhi (Cinnamomum cassia twig)	_
Hong 2006 [44]	Yiqiwenyanghuoxuetongluo Tang (Decoction)	Guizhi (Cinnamomum cassia twig), Xixin (Asarum heterotropoidesrhizhome)	_
Chen 2005 [45]	Yiqiwenyangtongluo Tang (Decoction)	Lujiaojiao (Cervus Nippon horn), Xianling pi (Herbaepimedii), Mahuang (Ephedra sinica stem), Shanzhuyu (Cornusofficinalis)	200 ml BID
Lin 2011 [46]	Zhiyu Tang (Decoction)	Shanzhuyu (Cornusofficinalis)	_
Yi 2008 [47]	Guilong Wan (Pills)	Guizhi (Cinnamomum cassia twig), Xixin (Asarum heterotropoidesrhizhome)	_
Li 2012 [48]	Guilong Wan (Pills)	Guizhi (Cinnamomum cassia twig), Xixin (Asarum heterotropoidesrhizhome)	
Chen 2009 [49]	Guilong Wan (Pills)	Guizhi (Cinnamomum cassia twig), Xixin (Asarum heterotropoidesrhizhome)	5 g TID
Huang 2015 [50]	Dangguisini Tang (Decoction)	Guizhi (Cinnamomum cassia twig), Xixin (Asarum heterotropoidesrhizhome), Ganjiang (Zingiber officinale)	-
Song 2015 [51]	Modified Guizhishaoyaozhimu Tang (Decoction)	Guizhi (Cinnamomum cassia twig), Ganjiang (Zingiber officinale), Fuzi (Aconitum carmichaeli root), Mahuang (Ephedra sinica stem),	200 ml BID
Chen 2015 [52]	Ruxiangyingtong Powder	Guizhi (Cinnamomum cassia twig), Shengjiang (Fresh Zingiber officinale)	200 ml TID
Zhao 2015 [53]	Yiqihuoxuetongluo Tang (Decoction)	Guizhi (Cinnamomum cassia twig), Xixin (Asarum heterotropoidesrhizhome), Fuzi (Aconitum carmichaeli root)	300 ml BID
Liu 2015 [54]	Modified Buyanghuanwu Tang (Decoction)	Huluba (Trigonellafoenum- graecum seeds), Haima (Hippocampus japonicas)	_
Ma 2015 [55]	Modified Dangguisini Tang (Decoction)	Guizhi (Cinnamomum cassia twig), Shengjiang (Fresh Zingiber officinale), Xixin (Asarum heterotropoidesrhizhome)	_
Chen 2015 [56]	Huangqiguizhiwuwu Tang (Decoction)	Rougui (Cinnamomum cassia bark), Xixin (Asarum heterotropoidesrhizhome)	_
Zhang 2015 [57]	Wenyangzhitong Tang (Decoction)	Guizhi (Cinnamomum cassia twig), Xixin (Asarum heterotropoidesrhizhome)	_
Lang 2015 [58]	Modified Huangqiguizhiwuwu Tang (Decoction)	Guizhi (Cinnamomum cassia twig), Shengjiang (Fresh Zingiber officinale)	100 ml BID

### Treatment effect

#### Treatment effect of Yang-warming Chinese medicine/yang-warming Chinese medicine plus western medicine compared with western medicine on peroneal nerve conduction velocity

**Fig. 2:**
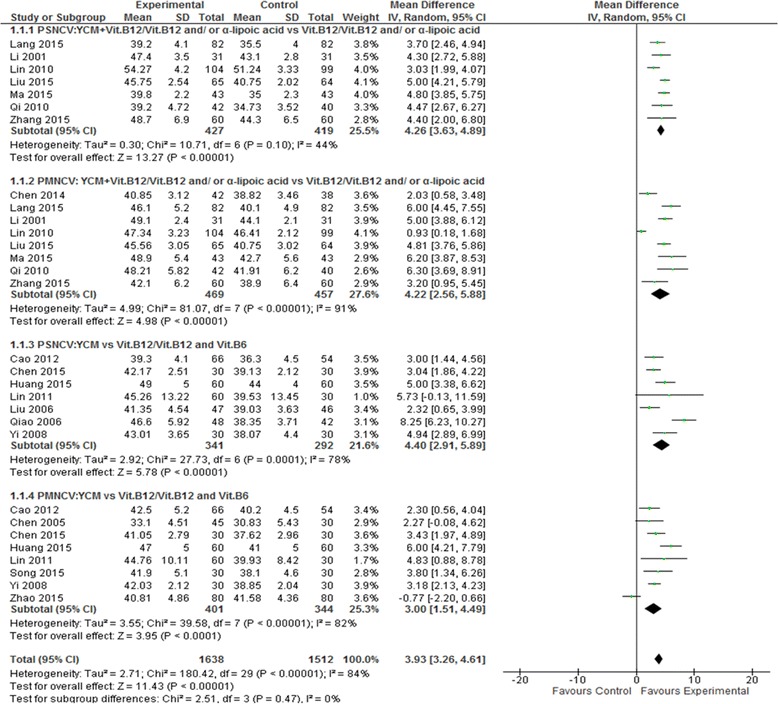
Forest plot of peroneal nerve conduction velocity of YCM on diabetic peripheral neuropathy. Note: PSNCV: peroneal sensory nerve conduction velocity; PMNCV: peroneal motor nerve conduction velocity

Peroneal nerve conduction velocity was observed in 18 of the 25 included studies including 10 YCM monotherapy studies [39, 40, 42, 45–47, 50–53] and 8 YCM combined with western medicine studies [34–36, 38, 54–56, 58]. Seven YCM monotherapy studies [39, 40, 42, 46, 47, 50, 52] compared the effect on peroneal sensory nerve conduction velocity with western medicine control (Vitamin B12/Vitamin B12 + B6) and 7 YCM combined with western medicine studies [35, 36, 38, 54, 55, 57, 58] compared the effect on peroneal sensory nerve conduction velocity with western medicine control (Vitamin B12/Vitamin B12 and/or α-Lipoic acid). The combined effect showed that YCM monotherapy had a significantly better effect on peroneal sensory nerve conduction velocity compared to western medicine control (*n* = 633, mean difference (MD) = 4.40, 95% CI [2.91, 5.89], *P* < 0.00001, heterogeneity chi-square = 27.73, *P* = 0.0001, I^2^ = 78%).YCM combined with western medicine was also significantly better in increasing peroneal sensory nerve conduction velocity compared with western medicine control (*n* = 846, mean difference (MD) = 4.26, 95% CI [3.63, 4.89], *P* < 0.00001, heterogeneity chi-square = 10.71, *P* = 0.10, I^2^ = 44%). Eight YCM monotherapy studies [42, 45–47, 50–53] compared the effect on peroneal motor nerve conduction velocity with western medicine control (Vitamin B12/Vitamin B12 + B6) and eight YCM combined with western medicine studies [34–36, 38, 54, 55, 57, 58] compared the effect on peroneal motor nerve conduction velocity with western medicine control (Vitamin B12/Vitamin B12 and/or α-Lipoic acid). The combined effect showed that both the therapy groups, YCM monotherapy and YCM combined with western medicine had a significantly better effect on peroneal motor nerve conduction velocity compared to western medicine control (*n* = 745, mean difference (MD) =3.00, 95% CI [1.51,4.49], *P* < 0.0001, heterogeneity chi-square = 39.58, *P* < 0.00001, I^2^ = 82%) and (*n* = 926, mean difference (MD) = 4.22, 95% CI [2.56, 5.88], *P* < 0.00001, heterogeneity chi-square = 81.07, *P* < 0.00001, I^2^ = 91%) respectively. (See Fig. 2).

#### Treatment effect of Yang-warming Chinese medicine/yang-warming Chinese medicine plus western medicine compared with western medicine on tibial nerve conduction velocity

**Fig. 3:**
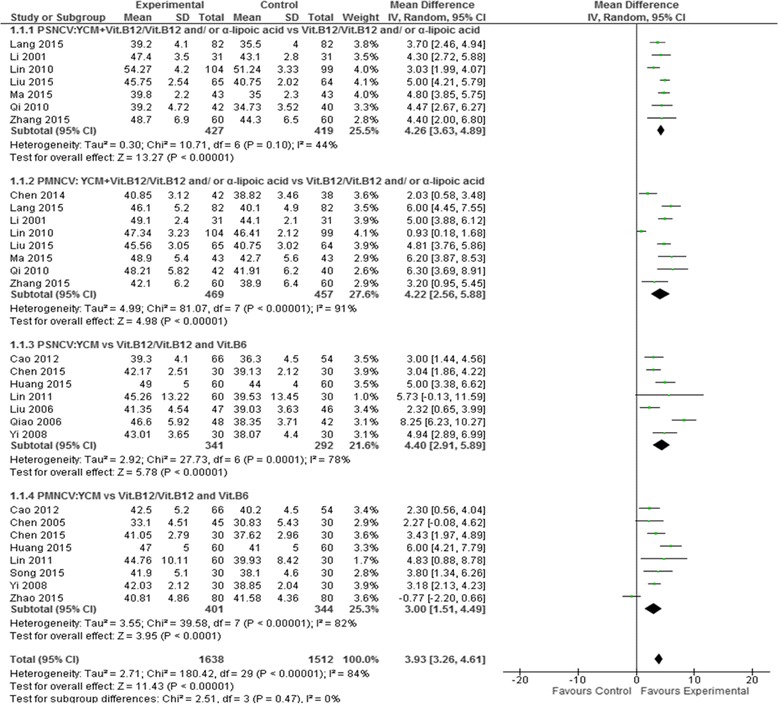
Forest plot of tibial nerve conduction velocity of YCM on diabetic peripheral neuropathy. Note: TSNCV: tibial sensory nerve conduction velocity; TMNCV: tibial motor nerve conduction velocity.

Tibial nerve conduction velocity was observed in 7 of the 25 included studies including 4 YCM monotherapy studies [39, 42, 45, 48] and 3 YCM combined with western medicine studies [34, 37, 56]. Two YCM monotherapy studies [39, 42] compared the effect on tibial sensory nerve conduction velocity with western medicine control (Vitamin B12/ Vitamin B12 + B6) and 2 YCM combined with western medicine studies [37, 56] compared the effect on tibial sensory nerve conduction velocity with western medicine control (Vitamin B12/Vitamin B12 + B1/Vitamin B12 and/or α-Lipoic acid). The combined effect showed that both the therapy groups, YCM monotherapy and YCM combined with western medicine had a significantly better effect on tibial sensory nerve conduction velocity compared to western medicine control (*n* = 210, mean difference (MD) = 3.51, 95% CI [1.34,5.69], *P* < 0.002, heterogeneity chi-square = 4.18, *P* = 0.04, I^2^ = 76%) and (*n* = 142, mean difference (MD) =3.20, 95% CI [2.17, 4.22] *P* < 0.00001, heterogeneity chi-square = 0.29, *P* = 0.59, I^2^ = 0%) respectively. Four YCM monotherapy studies [40, 43, 46, 51] and three YCM combined with western medicine studies [32, 35, 54] compared the effect on tibial motor nerve conduction velocity with western medicine control. The combined effect showed that YCM monotherapy was not favorable in increasing tibial motor nerve conduction velocity compared to western medicine (Vitamin B12/Vitamin B12 + B6) control (*n* = 475, mean difference (MD) =2.43, 95% CI [−0.17, 5.04], *P* = 0.07, heterogeneity chi-square = 22.79, *P* < 0.0001, I^2^ = 87%). YCM combined with western medicine was not favorable in increasing tibial motor nerve conduction velocity compared with western medicine (Vitamin B12/Vitamin B12 + B1/Vitamin B12 and/or α-Lipoic acid) control (*n* = 222, mean difference (MD) = 3.09, 95% CI [−0.38, 6.57]. (See Fig. 3).

#### Treatment effect of Yang-warming Chinese medicine/yang-warming Chinese medicine plus western medicine compared with western medicine on median nerve conduction velocity

**Fig. 4:**
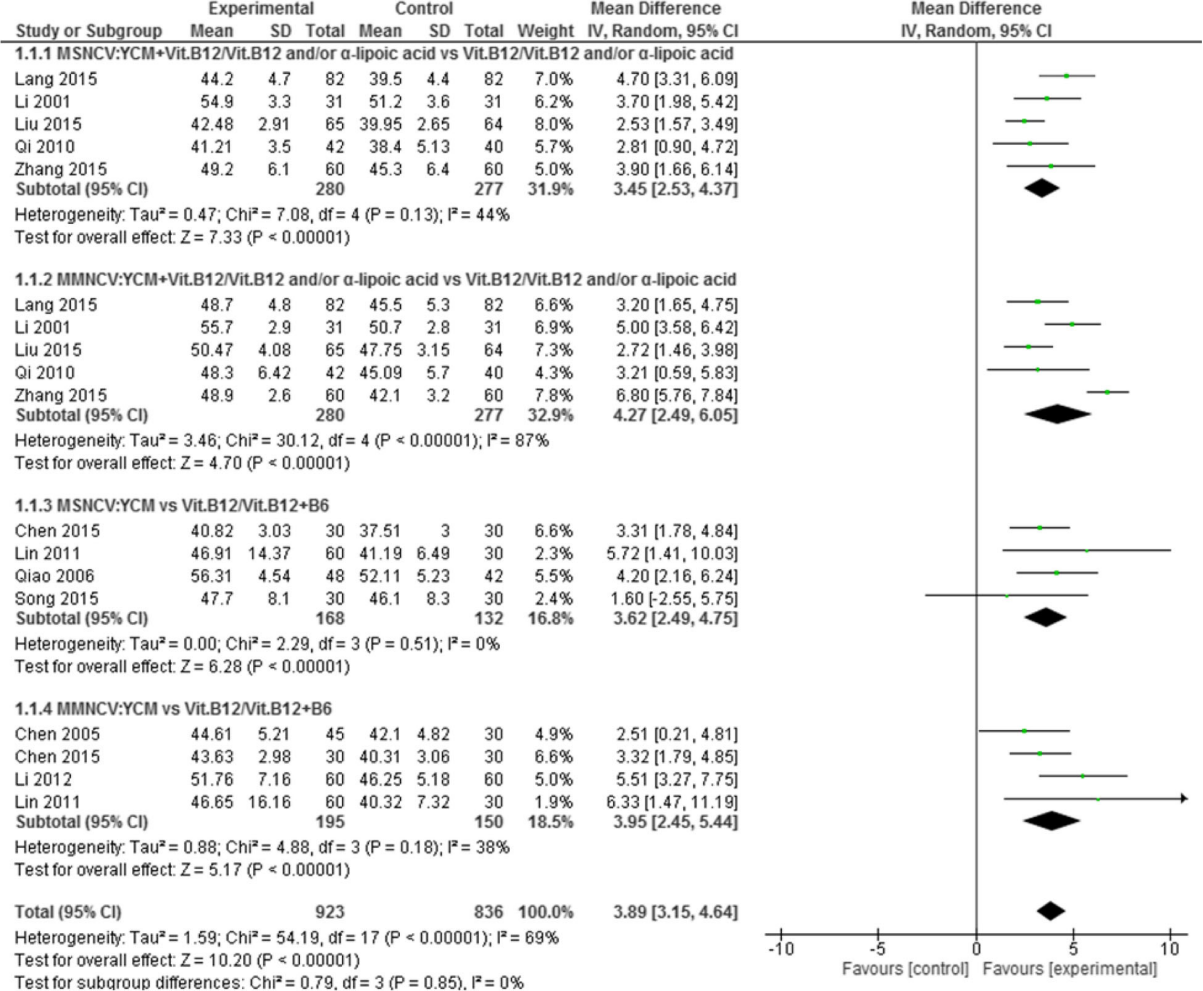
Forest plot of median nerve conduction velocity of YCM on diabetic peripheral neuropathy. Note: MSNCV: median sensory nerve conduction velocity; MMNCV: median motor nerve conduction velocity

Median nerve conduction velocity was observed in 11 of the 25 included studies including 6 YCM monotherapy studies [39, 45, 46, 48, 51, 52] and 5 YCM combined with western medicine studies [35, 36, 54, 57, 58]. Four YCM monotherapy studies [39, 46, 50, 52] compared the effect on median sensory nerve conduction velocity with western medicine control (Vitamin B12/Vitamin B12 + B6) and 5 YCM combined with western medicine study [35, 36, 54, 57, 58] compared the effect on median sensory nerve conduction velocity with western medicine control (Vitamin B12/Vitamin B12 and/or α-Lipoic acid). The combined effect showed that both the therapy groups, YCM monotherapy and YCM combined with western medicine had a significantly better effect on median sensory nerve conduction velocity compared to western medicine control (*n* = 200, mean difference (MD) = 3.62, 95% CI [2.49,4.75], *P* < 0.00001, heterogeneity chi-square = 2.29, *P* = 0.51, I^2^ = 0%) and (*n* = 557, mean difference (MD) =3.45, 95% CI [2.53, 4.37] *P* < 0.00001, heterogeneity chi-square = 7.08, *P* = 0.13, I^2^ = 44%) respectively. Four YCM monotherapy studies [45, 46, 48, 52] compared the effect on median motor nerve conduction velocity western medicine control (Vitamin B12/Vitamin B12 + B6) and five YCM combined with western medicine studies [35, 36, 54, 57, 58] compared the effect on median motor nerve conduction velocity with western medicine control(Vitamin B12/Vitamin B12 and/or α-Lipoic acid). The combined effect showed that YCM monotherapy had a significantly better effect on median motor nerve conduction velocity compared to western medicine control (*n* = 245, mean difference (MD) =3.95, 95% CI [2.45, 5.44], *P* < 0.00001, heterogeneity chi-square = 4.88, *P* = 0.18, I^2^ = 38%).YCM combined with western medicine was also significantly better in increasing median motor nerve conduction velocity compared with western medicine control (*n* = 557, mean difference (MD) =4.27, 95% CI [2.49, 6.05], *P* < 0.00001, heterogeneity chi-square = 1.38, *P* < 0.00001, I^2^ = 87%). (See Fig. 4).

#### Total efficacy rate of Yang-warming Chinese medicine /yang-warming Chinese medicine plus western medicine compared with western medicine

**Fig. 5:**
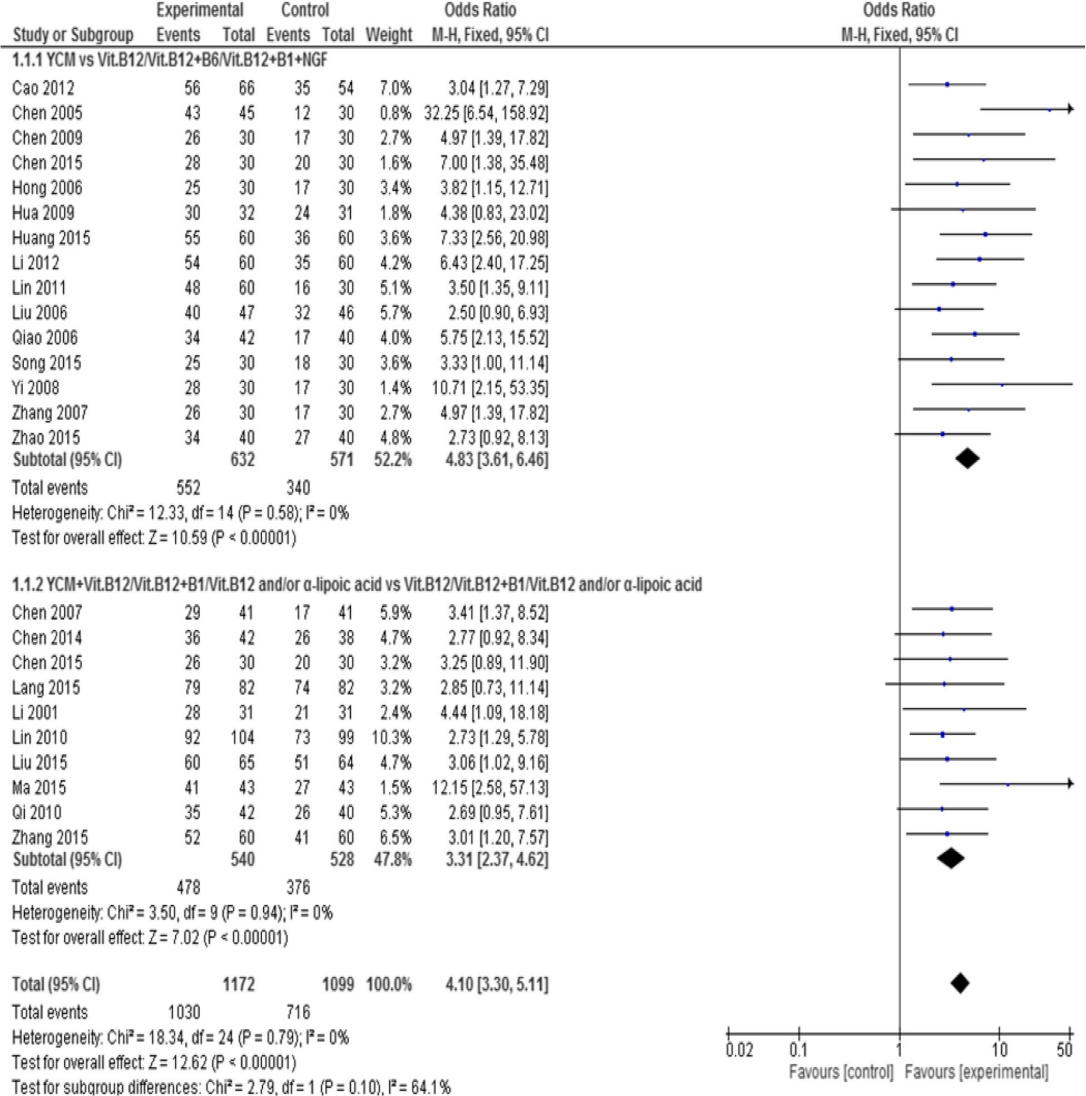
Forest plot of total efficacy rate of YCM on diabetic peripheral neuropathy

Total efficacy rate was accessed in all of the 25 included studies among which 15 were yang-warming Chinese medicine monotherapy [39–53] and 10 were yang warming Chinese medicine [34–38, 54–58] compared with western medicine control. Combined effect indicated that both the groups; Yang-warming Chinese medicine group and yang-warming Chinese medicine combined with western medicine group, were significantly better than western medicine alone in total efficacy rate (*n* = 1203, odds ratio (OR) = 4.83, 95% CI [3.61, 6.46], *P* < 0.00001, heterogeneity chi-square = 12.33, *P* = 0.58, I^2^ = 0%) and (*n* = 1068, odds ratio (OR) = 3.31, 95% CI [2.37, 4.62], *P* < 0.00001, heterogeneity chi-square = 3.50,*P* = 0.94, I^2^ = 0%) respectively. (See Fig. 5).

### Adverse events

Most of the included studies did not clearly reported about the adverse effects, however 5 studies [36, 39, 44, 48, 56] reported that there were no any adverse events and five studies [37, 42, 53, 54, 57] reported about the mild adverse effects. Chen 2007 [37] reported that one case in control group was found to have decreased white blood cells. Apart from that there were mild degree of abdominal pain, diarrhea, nausea, vomiting and other gastrointestinal reactions and were relieved after symptomatic treatment. Cao 2012 [45] reported that in treatment group, 3 cases had minor symptoms like thirst and frequent micturition, 2 cases had diarrhea and all the symptoms were disappeared after withdrawal of the medicine. Similarly in control group, 2 cases had symptoms of epigastric fullness and was relieved by domperidone. Due to the insufficient data, the specific circumstances of the drug adverse reactions were unclear, and so we failed to evaluate the adverse reactions of yang-warming Chinese medicine.

### Sensitivity analysis

Because of the poor quality of the 25 included studies, we were unable to analyze the sensitivity of the low quality literatures.

### Publication bias

**Fig. 6:**
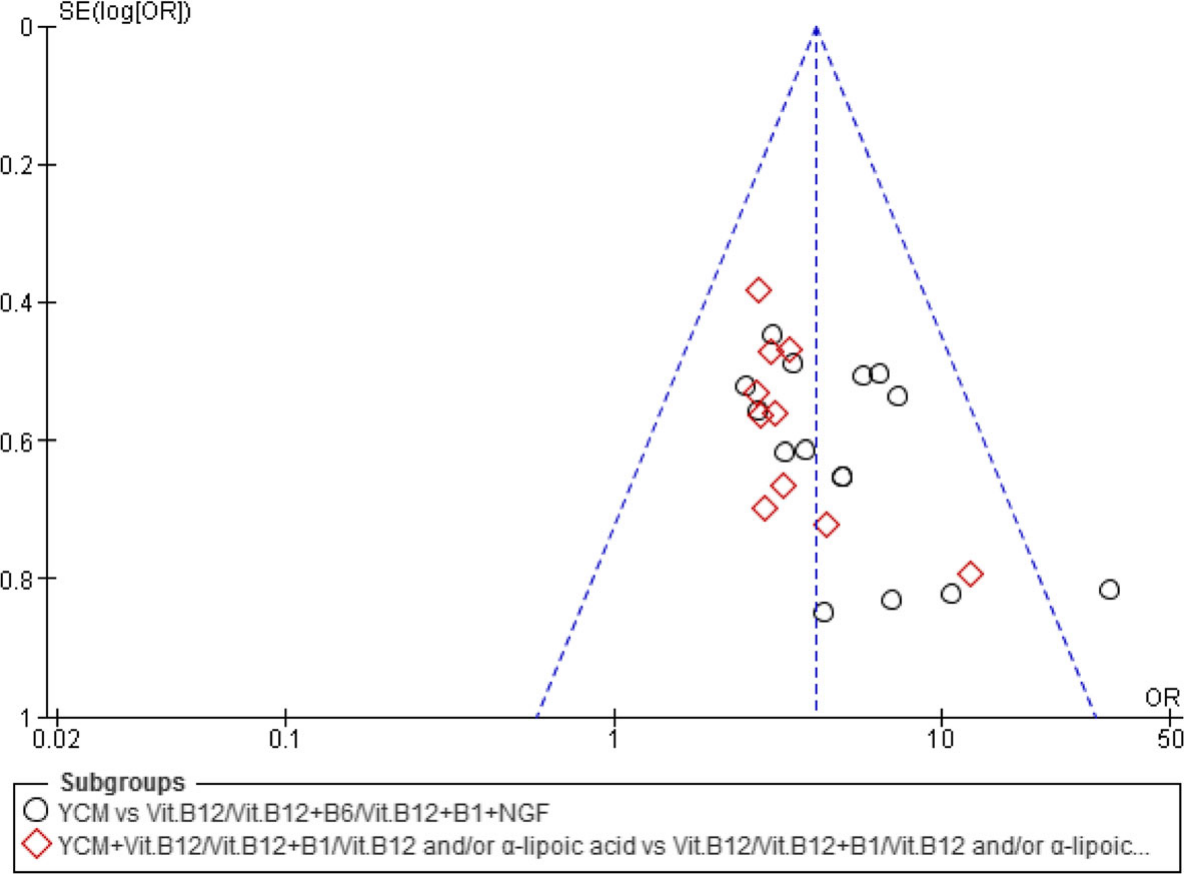
Funnel plot of total efficacy rate of YCM on diabetic peripheral neuropathy

The funnel plot analysis showed the evidence of publication bias (Fig. 6).

## Discussion

Our meta-analysis included 25 articles which applied yang-warming method in the treatment of diabetic peripheral neuropathy having clear diagnostic criteria, inclusion criteria and exclusion criteria. All the studies had pre-intervention group comparability, followed meta-analysis “prudent, accurate and sensible” thinking principle, its efficacy and safety system analysis. All the included studies were of low methodological quality scoring 1 to 2 in Jadad scale and 3–5 in 12- point criteria. Till date, there is no any meta-analysis reporting yang-warming treatment method of diabetic peripheral neuropathy.

### Clinical effectiveness

Our meta-analysis included total of 25 articles, of which 10 papers reported randomized control trials (RCTs) comparing combined western medicine and yang-waring Chinese medicine with western medicine alone; 15 papers reported RCTs comparing yang-warming Chinese medicine with western medicines in the treatment of diabetic peripheral neuropathy. Meta-analysis showed that: yang-warming Chinese medicine combined with western medicine was superior in increasing peroneal nerve, median nerve conduction velocity and better in clinical effectiveness compared to western medicine alone. Similarly yang-warming Chinese medicine group showed superiority in increasing peroneal nerve, tibial nerve, and median nerve conduction velocity and also in clinical efficacy compared to western medicine group. In short, yang-warming Chinese medicine treatment was an effective method in treating diabetic peripheral neuropathy.

### Clinical safety

A standard clinical trial should observe and mention whether there were adverse effects or not. Most of the included trials in our study did not clearly mentioned about the adverse effects and on the hand there were limited number of articles in our study. Ultimately, we failed to draw the conclusion that yang-warming Chinese medicine is safe for the treatment of diabetic peripheral neuropathy.

### Clinical research significance

Our Meta-analysis showed that the yang-warming method is effective in treating diabetic peripheral neuropathy, and it has a bright future, it is valuable to dig deep and provide more objective basis for the treatment of diabetic peripheral neuropathy. In the future, more and more scholars will be encouraged to carry out high quality, multi-level, multi-center, blinded randomized clinical trials with larger sample to further confirm its efficacy, improve patient quality of life so as to bring greater social economic benefits.

### Significance for clinical practice

This study confirmed the advantages of Chinese medicine treatment, unique advantages of Yang warming herbs in the treatment of diabetic peripheral neuropathy, and provides a new way of thinking to clinicians undertaking the development of traditional Chinese medicines. The treatment of diabetic peripheral neuropathy using yang-warming method has a good curative effect, it has broad prospects for development.

### Existing problems and prospects

#### Existing problems

i. Randomization method: Randomized controlled clinical trial is recognized as “gold standard” for evaluating the efficacy of certain intervention. In our study, three RCT literature clearly mentioned the use of a random number table, the remaining documents only mentioned the use of random method, but the specific method used is unknown. All the articles were rated as low grade articles. And moreover, the number of cases in the treatment group and the control group were different, so it can be considered as the unequal randomized control, which will reduce the effectiveness of test statistics. Therefore, how to do proper randomization in order to ensure the scientific nature of the randomized trials is one of the major issue.
ii. Allocation concealment scheme: None of the included articles in our study mentioned about the allocation concealment. Every standard RCTs should adopt proper allocation concealment scheme, otherwise test effect may be exaggerated.
iii. Lack of implementation of the blinding method: The objective of blinding is to effectively avoid researcher and subjective bias. A total of 16 documents included did not reported the use and the use of blinding. This may affect the test effect and characteristics of Chinese medicine.
iv. Withdrawal and dropouts: All the included studies did not mention about withdrawal and dropouts, will seriously affect the authenticity and credibility of the study findings.
v. Publication bias: The results of this analysis showed the publication bias. The reasons behind the emergence of bias may include sampling bias, selection bias, and bias within the studies.

#### Prospects

Due to the limited numbers of literatures in this meta-analysis, and moreover under the condition of improper design of the studies and their lower quality we could not carry out the sensitivity analysis. In the presence of statistical heterogeneity, we could not completely rule out the influence of clinical heterogeneity or methodological heterogeneity, so the results may be biased. Therefore, in future clinical studies, we can learn from the experience to carry out high-quality, multi-level, multi-center and properly blinded randomized controlled trials with large samples. In these types of trials, applying of the standard inclusion criteria, general diagnostic criteria, reasonable exclusion criteria, and high standard monitoring system could ensure the consistency of the measurement index units; would not exclude the negative results. The selection of high quality articles without neglecting withdrawal or dropouts and detail description of adverse effects could ensure the safety of clinical trials. All the trials included in our study were written in Chinese language and were published in china, so the findings of those studies could not be generalized. Therefore, in future there is a need to publish those studies in English language also so as to generalize the results. Due to the various limitations of our meta-analysis, the findings of our study should be interpreted with caution.

## Conclusion

Yang-warming Chinese medicines were apparently better than conventional western medicine alone in terms of nerve conduction velocity and clinical efficacy in treating DPN. However, due to the insufficient data available, we could not confirm the safety of yang-warming Chinese medicines. Because of the poor quality of the reported works that were available for the present meta-analysis, it is earlier to claim the superiority of yang-warming method using YCM to western medicines for the treatment of DPN. To support these early findings, further standardized and rigorous RCTs are required.

## Abbreviations

ADA: American diabetes association
AGEs: Advanced glycosylated end products
CI: Confidence interval
DM: Diabetes mellitus
DPN: Diabetic peripheral neuropathy
IGF: Insulin-like growth factor
MMNCV: Median motor nerve conduction velocity
MSNCV: Median sensory nerve conduction velocity
NCV: Nerve conduction velocity
PMNCV: Peroneal motor nerve conduction velocity
PSNCV: Peroneal sensory nerve conduction velocity
RCTs: Randomized controlled trials
SMD: Standardized mean difference
TCM: Traditional Chinese medicine
TMNCV: Tibial motor nerve conduction velocity
TSNCV: Tibial sensory nerve conduction velocity
WHO: World health organization
WHOPNTF: World health organization international collaborative research of the diabetic peripheral neuropathy
WMD/MD: Weighted mean difference/Mean difference
YCM: Yang-warming Chinese medicine
